# Human urinary extracellular vesicle preparations inhibit *in vitro* biofilm formation against several uropathogens

**DOI:** 10.3389/fmicb.2026.1782549

**Published:** 2026-03-11

**Authors:** Aziz Ur Rehman, Kieran Abbott, Fiona E. Karet Frankl, Ashraf Zarkan, Tim L. Williams

**Affiliations:** 1 Department of Veterinary Medicine, University of Cambridge, Cambridge, United Kingdom; 2 Department of Genetics, University of Cambridge, Cambridge, United Kingdom; 3 Cambridge Institute for Medical Research, University of Cambridge, Cambridge, United Kingdom

**Keywords:** antibiofilm activity, biofilms, urinary extracellular vesicles, urinary tract infections, uropathogens

## Abstract

**Introduction:**

Urinary tract infections (UTIs) rank as one of the most frequent bacterial infections globally, with multiple bacterial species such as uropathogenic *Escherichia coli* (UPEC), *Klebsiella pneumoniae*, and *Pseudomonas aeruginosa* being significant causative agents that can develop biofilms associated with antimicrobial resistance (AMR) and recurrence. Urinary extracellular vesicles (UEVs) are nanosized particles secreted by cells lining the urinary tract which carry nucleic acid and protein cargo, including antibacterial proteins, and high concentrations of UEVs exert antibacterial activity against UPEC *in vitro*. This study investigated the antibiofilm potential of UEVs against biofilm-forming uropathogens.

**Methods:**

UEV preparations from healthy human volunteers were added to bacteria, and biofilm formation was assessed using safranin-based biofilm quantification.

**Results:**

UEV preparations from the majority of volunteers significantly inhibited biofilm formation of multiple uropathogens, including UPEC (66.2% [34.8%–85.6%] inhibition vs. control), *P. aeruginosa* (37.2% [5.8%–42.6%]), and *K. pneumoniae* (31.8% [18.0%–60.4%]), an effect evident at physiologically relevant concentrations. UEV concentrations that exhibited antibiofilm activity were also not sufficient to inhibit bacterial growth.

**Discussion:**

These findings highlight the potential role of UEVs as innate modulators of uropathogen biofilms and lay the groundwork for future exploration of the relevance of host-derived UEVs in determining risks of recurrent UTIs.

## 1 Introduction

Urinary tract infections (UTIs) affect millions of people each year, with uropathogenic *Escherichia coli* (UPEC) responsible for 65%–75% of infections, although other bacterial species, including *Klebsiella pneumoniae* and *Pseudomonas aeruginosa*, are also causative agents (Medina and Castillo-Pino, 2019). Recurrence of UTIs occurs in 30%–40% of women (Kwok et al., 2022) and may be linked to the formation of biofilms (Murray et al., 2021). Biofilms form when bacteria adhered to surfaces release extracellular polymeric substances, which encapsulate and protect bacteria from the environment (Hancock et al., 2007), thus contributing to antimicrobial resistance (AMR) (Zhao et al., 2020). Hence, novel strategies for targeting biofilms require exploration.

Extracellular vesicles (EVs) are small, non-replicating, membrane-bound particles that are released by nearly all cell types and are found in different body fluids, including urine (Kumar et al., 2024). EVs carry a variety of molecular components that reflect the metabolic condition of the cells of their origin and are crucial for intercellular communication as well as exhibiting a range of functions that include genetic transfer, peptide transport, and antigen presentation (Buzas, 2023). Moreover, EVs display antibacterial potential, highlighting their role in host innate immunity (Timár et al., 2013). Human urinary extracellular vesicle preparations (UEVs) are enriched with bacteriostatic and bactericidal proteins and exhibit antibacterial activity against non-pathogenic and pathogenic *E. coli*, hence UEVs have been proposed as innate immune effectors of the urinary tract (Hiemstra et al., 2014). However, the impact of UEVs on the formation of biofilms is yet to be explored. Human respiratory epithelial cell-derived EVs prevent biofilm formation by *P. aeruginosa* by transferring EV-associated miRNA let-7b-5p into the bacterial cells (Koeppen et al., 2021), and let-7b-5p is also present in human UEVs (Gracia et al., 2017). Consequently, we hypothesized that UEVs would also prevent biofilm formation by uropathogens.

## 2 Methodology

### 2.1 Urine sample collection and quality assessment

The study was approved by HRA and Health Care Research Wales (IRAS Project ID 326042, REC reference 24/WA/0054). Urine samples were collected from healthy volunteers with their informed consent. Within 3 h of collection, dipstick analysis (Combur-Test, Roche Diagnostics) was performed, and samples with significant abnormalities were excluded from further analysis.

### 2.2 Urinary extracellular vesicles (UEV) preparation isolation

Following urinalysis, urine was centrifuged for 20 min at 17,000 × *g* at 4 °C in a high-speed centrifuge (Avanti JXN-26 Centrifuge; JA-25.50 rotor; Beckman Coulter). The pellet was discarded and the supernatant was passed through a 0.22 μm filter (Stericup, Millipore, Merck). The filtered supernatant was ultracentrifuged at 235,000 × *g* for 120 min at 4 °C with deceleration setting 7 (Optima XP-1N00 Ultracentrifuge; Type 45 Ti rotor; Beckman Coulter). The pellets from each tube were pooled together and suspended in 350 μL sterile Dulbecco’s Phosphate Buffered Saline (PBS; D8662, Sigma-Aldrich) prior to storage at −80 °C.

### 2.3 Transmission electron microscopy (TEM)

A total of 20 μL of UEV preparation were used to coat glow-discharged copper-carbon film grids (400 mesh; EM Resolutions) for 30 s. The grids were then washed twice with distilled water prior to negative staining with uranyl acetate for 30–45 s. Grids were air-dried and examined under an FEI Tecnai G^2^ electron microscope (Thermo Fisher Scientific Inc.) at the Cambridge Advanced Imaging Centre (Department of Anatomy, University of Cambridge, United Kingdom).

### 2.4 Western blotting

Samples were prepared by adding 10 μL of UEVs to 4x loading buffer with 0.3 M dithiothreitol (DTT). The protein samples were denatured at 75 °C for 5 min and then loaded onto a 4%–12% Bis-Tris polyacrylamide gel (1.5 mm thickness). A total of 5–7 μL of a full range molecular weight marker (28295341 RPN800E) was loaded alongside the sample for comparison. The gel was electrophoresed for 60 min at 170 V. Following electrophoresis, samples were transferred to a 0.45 μm nitrocellulose membrane (Whatman, Springfield) using a wet transfer system (20% methanol transfer buffer) at 30 V for 120 min. A total of 5% non-fat milk (Sigma-Aldrich) in 0.1% PBS-Tween 20 (PBST) was used to block the membrane for 60 min to prevent non-specific binding. The membrane was incubated overnight with two primary antibodies, purified mouse anti-TSG101 antibody (612696; BD Transduction Laboratories) and rabbit monoclonal anti-CD9 antibody (ab9276; abcam) at 1:1000 dilutions in 5% milk in 0.1% PBST at 4 °C. The membrane was then washed three times with 0.1% PBST (5 min each wash) and incubated with secondary antibodies, polyclonal rabbit anti-mouse HRP and goat anti-rabbit HRP antibody, in 1:5000 dilutions in 5% milk in 0.1% PBST for 60 min. Following incubation, the membrane was again washed three times. Peroxide and Luminol Enhancer Solution (Thermo Fisher Scientific Inc.) was added in a 1:1 ratio for 5 min for signal enhancement and then drained off. The membrane was then imaged using the Bio-Rad ChemiDoc imaging system in a darkroom.

### 2.5 Nanoparticle tracking analysis (NTA)

Urinary extracellular vesicle samples were characterized and quantified using a NanoSight NS300 Nanoparticle Tracking Analyzer (Malvern). UEVs were diluted between 1:200 and 1:20,000 in sterile Milli-Q water (Biopak; Merck Millipore) to achieve the analytical range of 30–80 particles per frame and injected into the chamber with a constant flow rate of 100 arbitrary units using the NanoSight syringe pump system (Malvern). The camera level was set to 15, and three videos of 60 s each were taken and analyzed at a detection threshold of six using NTA 3.2 software.

### 2.6 Antibiofilm assays

#### 2.6.1 Inoculation of bacterial strains in lysogeny broth (LB)

Strong biofilm-forming strains of *E. coli* (ATCC 25922), *P. aeruginosa* (PAO1), and *K. pneumoniae* (DSM 109314) were used for the antibiofilm assays. The bacteria were inoculated in autoclaved LB (Formedium Ltd.) in 30 mL polystyrene universal tubes (International Scientific Supplies Ltd.) prior to incubation in a shaking incubator (New Brunswick Innova 44R) for 18 h at 37 °C.

#### 2.6.2 Inoculation in artificial urine media (AUM)

Artificial urine media (AUM) was prepared based on the recipes of multipurpose artificial urine media and enhanced AU from the works of Sarigul et al. (2019), Rimbi et al. (2024), modified to more closely resemble the concentrations of metabolites in human urine, based primarily on the values from Bouatra et al. (2013). The most significant change is the inclusion of all canonical L-amino acids and a few D-amino acids, each at a concentration similar to that observed in human urine. The final concentrations of each component of artificial urine are shown in Supplementary Table 1. The artificial urine media was sterilized using a 0.22 μm minisart syringe filter (Sartorius AG), and 20 μL of inoculum, taken from overnight LB cultures, was added to 2 mL artificial urine media (100-fold dilution from the original inoculum) and incubated in a shaking incubator for 18 h at 37 °C.

#### 2.6.3 Plate setup

Overnight bacterial cultures were diluted in fresh artificial urine media to OD_600_ of 0.005 (approximately 4 million colony forming units/mL) measured using a spectrophotometer (GeneQuant™ 1300). A total of 180 μL of culture was added to Nunclon™ Delta Surface 96-well flat-bottom plates (ThermoScientific) for better cellular and biofilm adhesion. A total of 20 μL of UEV preparations or 20 μL of PBS (for controls) were added to each well, plates were covered with a moisture barrier seal and incubated in an IP100-U static incubator (LTE Scientific Ltd.) at 37 °C for 48 h.

#### 2.6.4 Safranin staining

Following incubation, the culture medium was carefully aspirated from the wells to remove the planktonic cells and to prevent the disruption or dislodgment of the attached biofilm. The wells were then washed twice using sterile PBS to remove any excess non-adherent cells and air-dried for 30 min at room temperature. A total of 250 μL of 0.25% safranin solution (Sigma-Aldrich) was added to each well using the Wellwash Versa microplate washer (ThermoScientific). The plate was incubated with the stain for 10 min, after which the stain was removed, and the plate was washed 10 times with distilled water using a set protocol in the microplate washer. The excess water was aspirated, and the plate was air-dried again for 60 min. A total of 200 μL of 30% acetic acid (Honeywell Research Chemicals) was added to each well and incubated for 30 min prior to transfer to flat-bottom 96-well plates (Falcon; Corning). Absorbance (corresponding to biofilm mass) was measured at 530 nm using a SpectraMax iD3 microplate reader (Molecular Devices). Given that *E. coli* is the predominant uropathogen responsible for UTIs, a more extensive replication strategy was implemented for the experiment. The safranin assay for *E. coli* experiment included UEV preparations, collected on different days, tested against three biological replicates of bacterial cultures, and each condition was analyzed in three technical replicates. For experiments involving other pathogens and *E. coli* dilution assays, a single UEV preparation of each volunteer was tested against three biological replicates of bacteria with two technical replicates per condition. The reduced number of UEV preparations in these experiments reflects practical constraints in vesicle yield and processing capacity. Differences in the biofilm inhibitory effect of UEV preparations across donors were due to inter-donor variations. Note that this method demonstrates biofilm formation and inhibition but does not differentiate whether the effect is due to reduced biomass, altered architecture, or impaired attachment.

### 2.7 Growth curve analysis

Bacterial cultures were grown in LB and artificial urine media using the protocol outlined previously. A total of 180 μL of culture (OD_600_ = 0.005) was added to flat-bottom 96-well plates (Falcon; Corning), and then either 20 μL of UEV preparations or 20 μL of PBS (for controls) was added to each well. The plate was incubated at 37 °C in a SpectraMax iD3 microplate reader, with absorbance at 600 nm (as a surrogate marker of bacterial number) measured every 10 min for 24 h.

### 2.8 Statistical analysis

All statistical analyses were performed using GraphPad Prism (v8.4.2). Data are reported as median [minimum–maximum]. Data were first assessed for normality using the Shapiro–Wilk test. As the data did not follow a normal distribution, non-parametric tests were applied. Comparisons between two groups were performed using the Mann–Whitney U test. Correlations were assessed using Spearman’s correlation. All tests were two-tailed, and a *p*-value < 0.05 was considered statistically significant. Statistical significance was denoted as: *P* < 0.05 (*), *P* < 0.01 (**), *P* < 0.001 (***), and *P* < 0.0001 (****).

## 3 Results

### 3.1 Urinalysis of collected urine samples

Urine samples were collected from healthy volunteers in multiple rounds. A total of 10 volunteers, six males and four females (aged 20–40), provided total first void samples on three separate days in the first cycle. For the second and third cycles of extractions, 130 mL of urine was collected from each volunteer. Urinalysis indicated trace amounts of protein, and pH was between 5 and 9, although most samples had pH 5–7. Occasional samples were trace positive for glucose and ketones; however, as the individuals were otherwise healthy, these were deemed likely false positives. The urinalysis findings for all isolations are presented in Supplementary Table 2.

### 3.2 Characterization of UEVs by TEM, Western blot, and NTA

**FIGURE 1:**
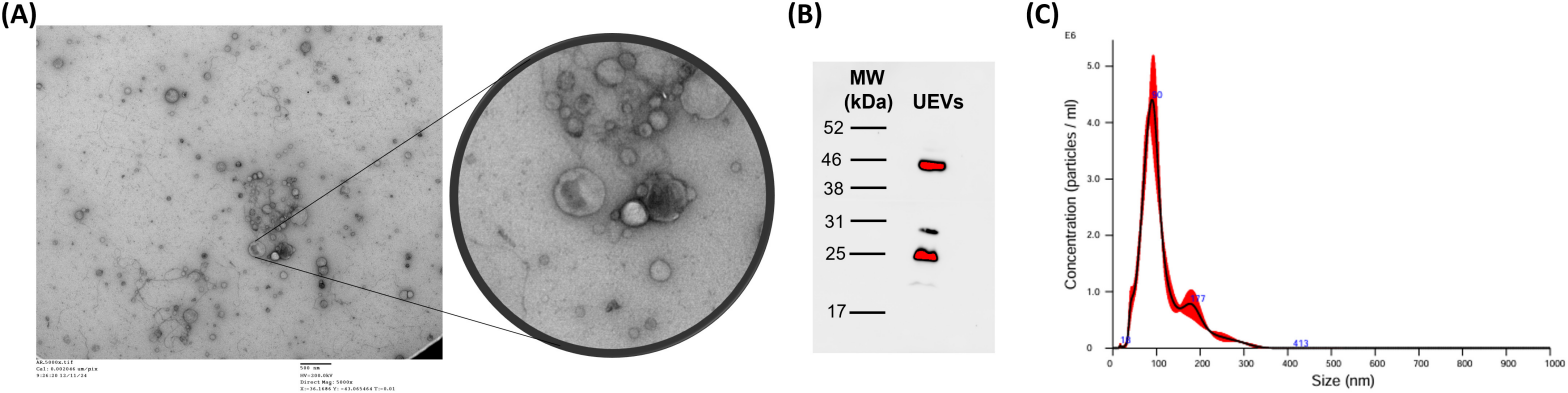
Characterization of urinary extracellular vesicles (UEVs). **(A)** Presence of UEVs is confirmed by visualization of their distinct cup-shaped morphology and 40–250 nm particle size. The sample from one of the volunteers was adsorbed on copper-carbon film grids, using uranyl acetate for negative staining, and observed under transmission electron microscopy (TEM) at × 5,000 direct magnification (200 kV). Scale bar: 500 nm. **(B)** Detection of characteristic EV surface markers by Western blot. The bands confirm the presence of respective UEVs markers, TSG101 (∼46 kDa) and CD9 (∼25 kDa). **(C)** Nanoparticle tracking analysis (NTA) results show peak distribution between the range of 40–250 nm particle size, indicating the presence of UEVs.

Expected UEV morphology (cup-shaped with bilayer membrane) and size (40–250 nm) was shown by TEM (Figure 1A; Li et al., 2022; Musante et al., 2020; Pisitkun et al., 2004).

Western blot confirmed the presence of canonical EV markers, TSG101 and CD9 (Figure 1B; Filipović et al., 2024; Musante et al., 2020). The NTA analysis of UEV samples indicated particle size distribution 50–250 nm, within the reported size range for UEVs (Figure 1C; Musante et al., 2020).

### 3.3 Screening of UEV preparations in antibiofilm assays

#### 3.3.1 Biofilm Inhibition against *E. coli* (ATCC 25922)

**FIGURE 2:**
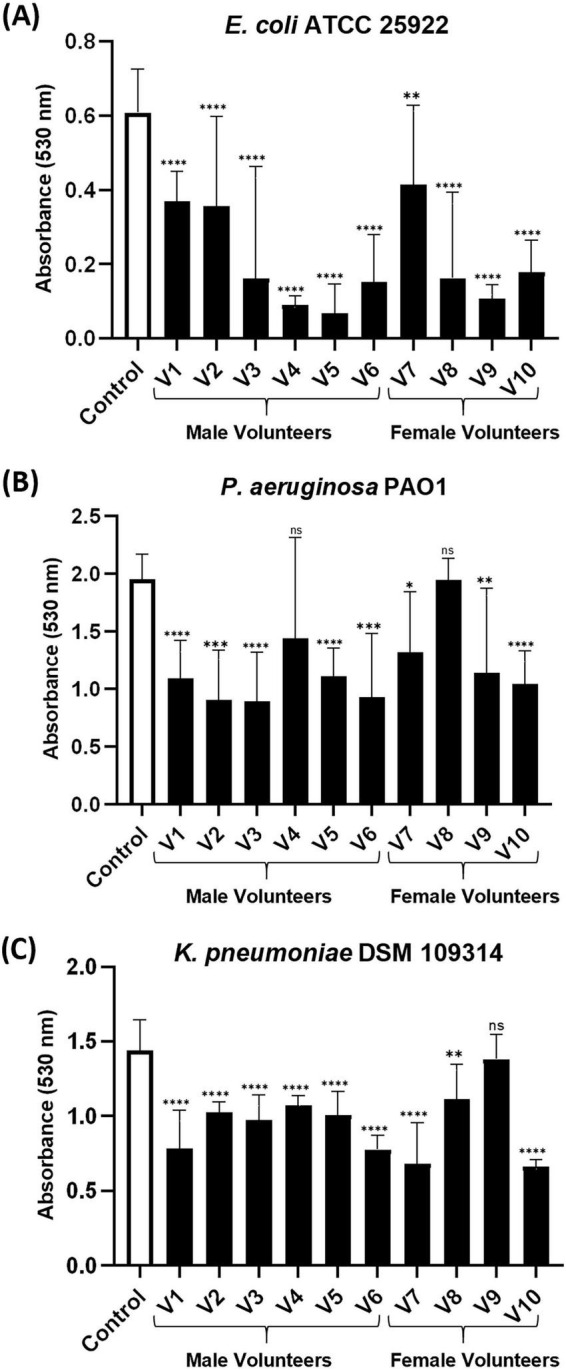
Screening of urinary extracellular vesicles (UEVs) for antibiofilm activity. **(A)** Inhibition of *E. coli* (ATCC 25922) biofilm formation by UEV preparations from 10 volunteers. The assay included three biological replicates of UEV preparations combined with bacteria, each analyzed in triplicates. **(B)** UEV preparations from 8 out of 10 healthy volunteers significantly reduced *P. aeruginosa* (PAO1) biofilm formation. **(C**) UEV preparations from 5/6 volunteers significantly reduced *K. pneumoniae* (DSM 109314) biofilm formation. The absorbance on the y-axis is biomass dependent and indicates biofilm formation; the x-axis represents control (no UEVs) and biofilms grown with UEV preparations of respective volunteers (V1–V10). Bars represent median ± IQR. Statistical comparisons were made using the Mann-Whitney U test, comparing each UEV-treated condition to the untreated control. Significance is indicated as: *P* < 0.05*, *P* < 0.01**, *P* < 0.001***, and *P* < 0.0001****.

Urinary extracellular vesicle preparations from all 10 volunteers showed significant inhibition of biofilm formation by *E. coli* (66.2% [34.8%–85.6%] inhibition vs. control; Figure 2A).

#### 3.3.2 Biofilm inhibition against *P. aeruginosa* (PAO1)

Urinary extracellular vesicle preparations from eight volunteers significantly reduced biofilm formation compared to controls (37.2% [5.8%–42.6%] inhibition vs. control; Figure 2B).

#### 3.3.3 Biofilm inhibition against *K. pneumoniae* (DSM 109314)

Urinary extracellular vesicle preparations from nine volunteers demonstrated a significant reduction in biofilm formation relative to the untreated control (31.8% [18.0%–60.4%] inhibition vs. control; Figure 2C).

### 3.4 Correlation between UEV concentration and biofilm inhibition

**FIGURE 3:**
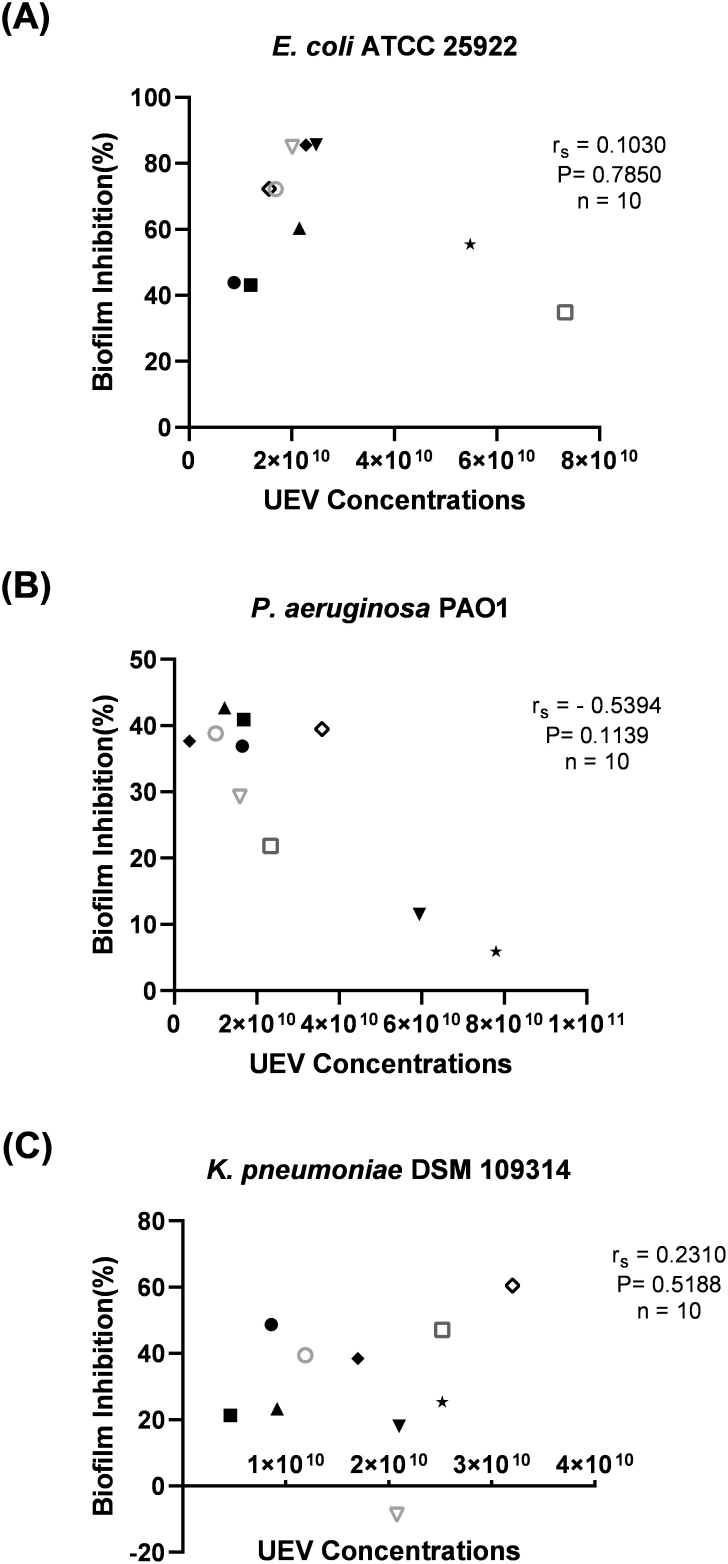
Correlation analysis between urinary extracellular vesicles (UEV) concentration and percentage biofilm inhibition. **(A)** For *E. coli*, no correlation was observed (r_*s*_ = 0.1030, *P* = 0.7850). **(B)** *P. aeruginosa* biofilm inhibition was not significantly associated with UEV concentrations (r_*s*_ = –0.5394, *P* = 0.1139). **(C)** No significant correlation was observed between UEV concentration and percentage biofilm inhibition of *K. pneumoniae* (r_*s*_ = 0.2310, *P* = 0.5188). Each point represents the UEV concentration of the UEV preparation from a different individual donor sample (*n*). Correlation coefficients (r_*s*_) and two-tailed *P*-values are indicated for each species.

In order to evaluate if the inhibition of biofilm was influenced by UEV concentrations within the UEV preparations isolated from different individuals, UEV concentration (in the experiment) was correlated with percentage biofilm inhibition for *E. coli*, *P. aeruginosa*, and *K. pneumoniae*. None of the correlations reached statistical significance (*P* > 0.05), indicating no strong relationship between UEV concentration and biofilm inhibition in the current dataset (Figure 3).

### 3.5 Dose-dependent antibiofilm activity of UEVs

Urinary extracellular vesicle preparations of six volunteers (three males, three females) from the original cohort were diluted 10-fold (corresponding to a 3.7-fold increase in UEV concentrations relative to UEV concentrations in urine) and 100-fold (corresponding to ∼3-fold dilution of UEV concentrations relative to concentrations in urine) to assess for dose-dependent antibiofilm activity.

#### 3.5.1 Against *E. coli* (ATCC 25922)

**FIGURE 4:**
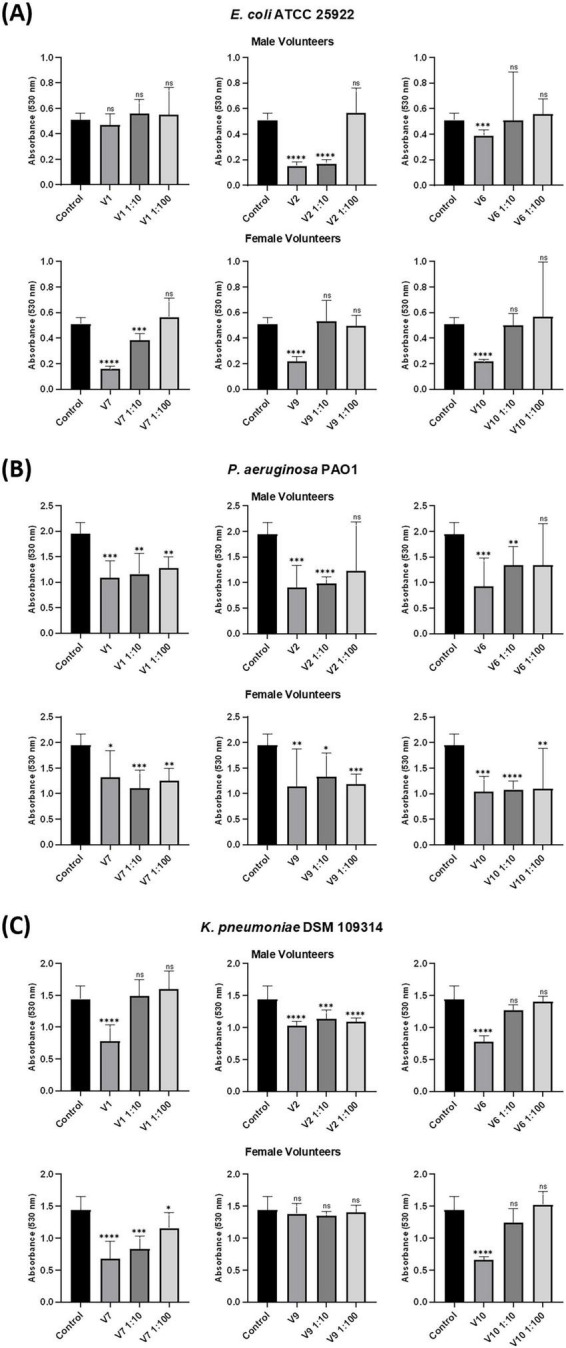
Dose-dependent inhibition of biofilm formation. Additional samples from three male and three female healthy volunteers were obtained. **(A)** Antibiofilm activity against *E. coli* (ATCC 25922). Among the six samples assessed, five showed inhibitory effects at undiluted concentrations, with 2 out of 3 males and all 3 out of 3 females demonstrating this response. Urinary extracellular vesicles (UEV) preparations from 1 out of 3 males and 1 out of 3 females also exhibited significant inhibition at a 10-fold dilution; however, no significant inhibition was observed from UEV preparations in any of the volunteers at the 100-fold dilution. **(B)** Against *P. aeruginosa* (PAO1), UEV preparations from all participants significantly reduced biofilm at undiluted and at 10-fold diluted concentrations. Among the participants, 1/3 male and all the females exhibited significant inhibition at a 100-fold dilution. **(C)** Five out of 6 UEV preparations significantly inhibited biofilm formation of *K. pneumoniae* (DSM 109314) at undiluted concentration, while 2 out of 6 (1 male, 1 female) showed significant inhibition at both 10-fold and 100-fold dilutions. Bars represent median ± IQR of three biological and two technical replicates. The Mann-Whitney U test was performed to compare the control and the UEV-treated wells data. Significance levels are indicated as: *p* < 0.05*, *p* < 0.01**, *p* < 0.001***, *p* < 0.0001****.

Out of the six tested samples, five demonstrated significant inhibition at undiluted concentration (55.8% [11.6%–68.8%] inhibition vs. control), including 2/3 male and all 3/3 female volunteers. UEVs from 1/3 males and 1/3 females also caused significant inhibition at a 10-fold dilution; however, no inhibition was noted by UEV preparations from any volunteers at the 100-fold dilution (Figure 4A).

#### 3.5.2 Against *P. aeruginosa* (PAO1)

Urinary extracellular vesicle preparations from all participants prevented biofilm formation at undiluted concentrations (37.8% [21.8%–40.8%] inhibition compared to control) (Figure 4B). All six samples also demonstrated antibiofilm activity at a 10-fold dilution. Among the volunteers, one out of three males and all females displayed significant inhibition of biofilm formation at a 100-fold dilution.

#### 3.5.3 Against *K. pneumoniae* (DSM 109314)

Five out of six UEV preparations significantly inhibited biofilm formation at undiluted concentrations (43.3% [− 8.7%–60.4%] inhibition compared to the control), while two out of six (one male, one female) showed inhibition at both 10-fold and 100-fold dilutions (Figure 4C).

### 3.6 Growth curve analysis

**FIGURE 5:**
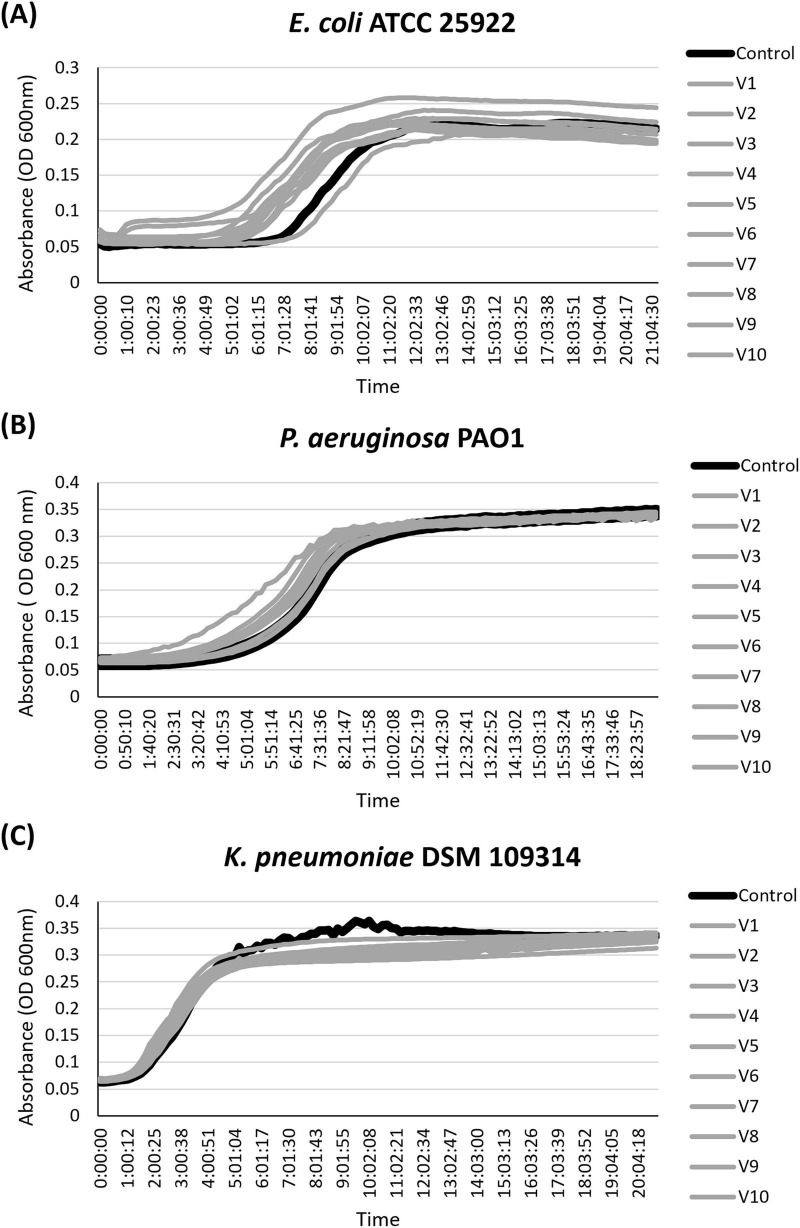
Growth curve analysis. Urinary extracellular vesicles (UEV) preparations were tested against **(A)** *E. coli*, **(B)** *P. aeruginosa*, and **(C)** *K. pneumoniae*, using undiluted UEV preparations. Bacteria were grown in artificial urine media with a starting inoculum of OD_600_ 0.005. UEV-treated growth curves were not significantly different to those of controls for all three bacterial species, confirming that the antibiofilm activity of UEV preparations is not secondary to an antibacterial effect of UEVs. Each curve represents the mean of OD 600 nm taken every 10 min for 24 h.

Growth curve assays were performed for *E. coli*, *P. aeruginosa*, and *K. pneumoniae* to evaluate whether the concentrations of UEVs associated with antibiofilm properties were associated with an antibacterial effect. All three bacterial species were grown in the presence of undiluted UEV preparations from each volunteer, at the same concentration that inhibited biofilm formation. No significant reduction in growth rate was observed between the UEV-treated bacteria and control conditions (Figure 5). The longer duration of the lag phase observed was attributed to the use of nutrient-deficient media (i.e., artificial urine), as well as a low starting inoculum size.

## 4 Discussion

Cells lining the urinary tract secrete UEVs, which have been investigated for their diverse functions, including their antibacterial activity and putative role as innate immune effectors of the urinary tract (Hiemstra et al., 2014). This study focuses on a novel aspect, namely the antibiofilm activity of UEV preparations isolated from healthy human individuals.

The antibiofilm activity of UEV preparations was robust across replicates and, although variable in efficacy between individuals and biological replicates, was demonstrated across common relevant bacterial uropathogens, namely *E. coli*, *P. aeruginosa*, and *K. pneumoniae.* The ability of UEV preparations to prevent biofilm formation suggests another mechanism by which UEVs help to prevent urinary tract infections. The antibiofilm effect of UEV preparations derived from individual volunteers also exhibited a dose-dependent response and, in most individuals, was demonstrated at UEV concentrations consistent with those found in urine, supporting the premise that this is a biologically relevant finding.

Hiemstra et al. (2014) showed that UEV preparations exhibited bactericidal activity against uropathogenic *E. coli* (UPEC), resulting in both growth inhibition and bacterial lysis, although the concentrations of UEVs that are associated with bactericidal activity were higher than those used in the present study. Proteomic investigations demonstrated that UEV preparations are enriched in a range of bactericidal and bacteriostatic proteins, which are hypothesized to be the effectors for the antimicrobial effects of UEVs. The mechanism of action of UEVs against biofilm formation has yet to be evaluated, although it is likely that this can also be attributed to the UEV cargo, perhaps specific proteins or miRNAs. In support of this, human respiratory epithelial cells produce EVs that inhibit biofilm formation by *P. aeruginosa* through the action of the miRNA let-7b-5p, which reduces the expression of proteins involved in biofilm formation and increases antimicrobial susceptibility (Koeppen et al., 2021). Previous studies from our group have confirmed the presence of let-7b-5p within human UEV preparations that were prepared using the same protocol to that of the present study (Gracia et al., 2017), although abundance of let-7b-5p was not assessed in our UEV preparations. Furthermore, engineered EVs incorporating let-7b-5p inhibited biofilm formation (Sarkar et al., 2025), supporting a potential role of let-7b-5p within UEVs in determining their antibiofilm activity, reinforcing the hypothesis that UEVs exert similar effects on uropathogens. Feasibly, the lack of correlation between UEV concentrations and antibiofilm activity could reflect variations in the abundance of let-7b-5p (or another effector molecule) between individual UEVs, hence further studies to establish if the abundance of let-7b-5p within UEVs is associated with UEV antibiofilm activity are warranted.

These findings are supported by emerging evidence across different biological contexts that EVs can modulate bacterial activity in diverse and often selective ways. For example; prostate-derived EVs are antibacterial against *Bacillus spp.* and *Pneumococcus spp* (Carlsson et al., 2000), EVs derived from human granulocytes have antibacterial activity against *Staphylococcus) aureus* (Timár et al., 2013), EVs released by tracheobronchial ciliated epithelial cells and airway cells neutralize human influenza virus (Kesimer et al., 2009), and EVs derived from oral mucosal cells demonstrate antimicrobial activity against *Candida albicans* (Zhao et al., 2022). In addition, a recent paper identified that EVs derived from human monocytes demonstrated antibiofilm activity against *Pseudomonas aeruginosa* (Solomon et al., 2025). The mechanism of action of EVs against different pathogens is likely to be variable, but a unifying theme is the protein and nucleic acid cargo within UEVs. Biofilm formation by uropathogens plays a key role in chronic UTIs and their recurrence, as it enables bacterial persistence, immune evasion, and is a major contributor to antibiotic resistance (Zhao et al., 2020). At physiological concentrations of UEVs, antibiofilm activity was demonstrated (without any antibacterial effect), which could indicate that UEVs help to prevent the formation of biofilms that might otherwise perturb the normal commensal urinary microbiome in healthy individuals (Perez-Carrasco et al., 2021). Since mature biofilms are difficult to eradicate, preventing early biofilm formation would be a biologically effective approach to preventing biofilm-associated infections in the urinary tract. The activity of UEV preparations against already established biofilms was not evaluated as part of this investigation, however would be of benefit to identify if UEVs might also play a role in eradicating biofilm-associated infections.

The use of artificial urine media for biofilm growth, along with the inhibition associated with UEVs at near-physiological concentrations, suggests that this phenomenon could occur *in vivo* within the urinary tract of healthy individuals. Concentrations of UEVs that exhibited antibiofilm activity did not affect the planktonic growth of bacteria, indicating that the inhibition of biofilm is not a consequence of a bactericidal action, but instead may involve specific disruption of the pathways responsible for biofilm formation. These results highlight that, apart from their role as cellular messengers, the functions of UEVs can be hypothesized to be involved in maintaining urinary tract sterility, possibly by disrupting early-stage biofilm formation, a critical step in the pathogenesis of UTIs. These findings collectively provide strong evidence for the bioactivity of UEVs and establish a basis for further studies to evaluate the relevance of UEVs in predisposition to UTIs and recurrent UTIs.

While our work provides compelling evidence for the antibiofilm activity of UEVs, some limitations require acknowledgment. First, the isolation protocol employs ultracentrifugation which, while practical, lacks specificity and will co-isolate other substances, most notably uromodulin (Furi et al., 2017), hence we refer to UEV preparations when describing our results in this manuscript. Future investigations might benefit from adding further purification steps, such as size exclusion chromatography (SEC), to enhance UEV purity prior to testing antibiofilm activity. Second, NTA was employed for UEV quantification, and although useful for estimating particle size and concentration, NTA cannot discriminate between EVs and other particles of similar size (e.g., protein aggregates) (Kowkabany and Bao, 2024), which could account for the lack of correlation between UEV concentration and percentage biofilm inhibition. Furthermore, whilst we investigated the antibiofilm properties of UEVs against various individual uropathogens, we only evaluated laboratory strains, which might not fully recapitulate the phenotypes of clinical strains, plus *in vivo* biofilms typically consist of multiple species, therefore, future evaluation of the antibiofilm activity of UEV preparations in a multispecies biofilm model (Nzakizwanayo et al., 2019) would be beneficial to provide further support for the biological relevance of these findings. In addition, the number of healthy volunteers included in this study may have been inadequate to capture the full spectrum of inter-individual variation in UEV composition and function based on parameters such as sex and ethnicity.

## 5 Conclusion

This study highlights the potential capability of UEVs to serve as natural inhibitors of uropathogenic biofilm formation. UEV preparations consistently inhibited biofilm formation by uropathogens at concentrations that were not associated with antibacterial activity suggesting that host-derived UEVs play a role in innate mucosal defense by modifying bacterial behavior rather than by eliminating bacteria. Ultimately, a deeper understanding of the mechanism of this activity would be beneficial and might help to leverage novel strategies for treating and preventing urinary tract infections.

## Data Availability

The raw data supporting the conclusions of this article will be made available by the authors, without undue reservation.
