# Supplementary material for: Human urinary extracellular vesicle preparations inhibit in vitro biofilm formation against several uropathogens
Source: Front Microbiol. 2026 Mar 11;17:1782549. doi: 10.3389/fmicb.2026.1782549 (PMC13013504; doi:10.3389/fmicb.2026.1782549)
Supplement: Supplementary file 1 [file Table_1.docx]

**Supplementary Table 1: Chemical composition of artificial urine media.**

| **Name** | **Concentration** |
| --- | --- |
| Sodium sulfate | 12.0 mM |
| Citric acid | 3.3 mM |
| Trisodium citrate dihydrate | 2.5 mM |
| Creatinine | 7.8 mM |
| Urea | 249.8 mM |
| Potassium chloride | 31.0 mM |
| Sodium chloride | 30.0 mM |
| Calcium chloride | 1.7 mM |
| Ammonium chloride | 23.7 mM |
| Potassium oxalate monohydrate | 0.2 mM |
| Magnesium sulfate heptahydrate | 4.4 mM |
| Sodium phosphate monobasic anhydrous | 18.7 mM |
| Sodium phosphate dibasic anhydrous | 4.7 mM |
| Uric acid | 1.5 mM |
| Hippuric acid | 3.5 mM |
| Thiamine-HCl | 0.003 mM |
| L-Cysteine | 1000.0 µM |
| L-Tyrosine | 150.0 µM |
| L-Galactose | 100.0 µM |
| L-Glucose | 500.0 µM |
| L-Sorbitol | 100.0 µM |
| L-Carnitine | 65.0 µM |
| L-Citrulline | 10.0 µM |
| L-Ornithine | 65.0 µM |
| D-Alanine | 35.0 µM |
| D-Aspargine | 15.0 µM |
| D-Serine | 125.0 µM |
| L-Alanine | 290.0 µM |
| L-Arginine | 110.0 µM |
| L-Asparagine | 130.0 µM |
| L-Aspartic acid | 150.0 µM |
| L-Glutamic acid | 100.0 µM |
| L-Glutamine | 520.0 µM |
| L-Glycine | 1400.0 µM |
| L-Histidine | 600.0 µM |
| L-Isoleucine | 20.0 µM |
| L-Leucine | 40.0 µM |
| L-Lysine | 240.0 µM |
| L-Methionine | 15.0 µM |
| L-Phenylalanine | 90.0 µM |
| L-Proline | 15.0 µM |
| L-Serine | 310.0 µM |
| L-Threonine | 190.0 µM |
| L-Tryptophan | 80.0 µM |
| L-Valine | 60.0 µM |

**Supplementary Table 2: Summary of urinalysis results for all volunteers across different extraction rounds.**

| Extraction Round | Volunteer No. | Volume (mL) | Glucose | Bilirubin | Ketones | Specific Gravity | Blood | pH | Protein | Urobilinogen | Nitrite | Leucocytes |
| --- | --- | --- | --- | --- | --- | --- | --- | --- | --- | --- | --- | --- |
| First Round | V1 (Day 1) | 260 | - | - | - | 1.026 | - | 5.0 | Trace | - | - | - |
|  | V1 (Day 2) | 380 | - | - | - | 1.016 | - | 5.0 | Trace | - | - | - |
|  | V1 (Day 3) | 350 | - | - | - | 1.020 | - | 5.0 | Trace | - | - | - |
|  | V2 (Day 1) | 305 | - | - | - | 1.010 | - | 5.0 | Trace | - | - | - |
|  | V2 (Day 2) | 230 | - | - | - | 1.012 | - | 5.0 | Trace | - | - | - |
|  | V2 (Day 3) | 290 | - | - | - | 1.008 | - | 5.0 | Trace | - | - | - |
|  | V3 (Day 1) | 195 | - | - | - | 1.020 | - | 5.0 | Trace | - | - | - |
|  | V3 (Day 2) | 195 | - | - | - | 1.016 | - | 5.0 | Trace | - | - | - |
|  | V3 (Day 3) | 195 | - | - | - | 1.012 | - | 5.0 | Trace | - | - | - |
|  | V4 (Day 1) | 260 | Trace | - | - | 1.006 | - | 5.0 | Trace | - | - | - |
|  | V4 (Day 2) | 195 | - | - | Trace | 1.010 | - | 5.0 | Trace | - | - | - |
|  | V4 (Day 3) | 130 | Trace | - | Trace | 1.004 | - | 5.0 | Trace | - | - | - |
|  | V5 (Day 1) | 65 | - | - | - | 1.020 | - | 7.0 | Trace | - | - | - |
|  | V5 (Day 2) | 195 | - | - | - | 1.020 | - | 5.0 | Trace | - | - | - |
|  | V5 (Day 3) | 195 | - | - | - | 1.020 | - | 5.0 | Trace | - | - | - |
|  | V6 (Day 1) | 195 | - | - | - | 1.008 | - | 5.0 | Trace | - | - | - |
|  | V6 (Day 2) | 130 | - | - | - | 1.016 | - | 5.0 | Trace | - | - | - |
|  | V6 (Day 3) | 130 | - | - | - | 1.020 | - | 5.0 | Trace | - | - | - |
|  | V7 (Day 1) | 220 | - | - | - | 1.040 | - | 5.0 | Trace | - | - | - |
|  | V7 (Day 2) | 210 | - | - | - | 1.040 | - | 5.0 | Trace | - | - | - |
|  | V7 (Day 3) | 255 | - | - | - | 1.012 | - | 5.0 | Trace | - | - | - |
|  | V8 (Day 1) | 240 | - | - | - | 1.016 | - | 5.0 | Trace | - | - | - |
|  | V8 (Day 2) | 225 | - | - | - | 1.012 | - | 5.0 | Trace | - | - | - |
|  | V8 (Day 3) | 185 | - | - | - | 1.012 | - | 5.0 | Trace | - | - | - |
|  | V9 (Day 1) | 195 | - | - | - | 1.012 | - | 5.0 | Trace | - | - | - |
|  | V9 (Day 2) | 130 | - | - | - | 1.020 | - | 5.0 | Trace | - | - | - |
|  | V9 (Day 3) | 65 | - | - | - | 1.024 | - | 5.0 | Trace | - | - | - |
|  | V10 (Day 1) | 195 | - | - | Trace | 1.016 | - | 5.0 | Trace | - | - | - |
|  | V10 (Day 2) | 195 | - | - | - | 1.016 | - | 5.0 | Trace | - | - | - |
|  | V10 (Day 3) | 195 | - | - | - | 1.010 | - | 5.0 | Trace | - | - | - |
| Second Round | V1 | 130 | - | - | - | 1.016 | - | 5.0 | Trace | - | - | - |
|  | V2 | 130 | - | - | - | 1.016 | - | 5.0 | Trace | - | - | - |
|  | V3 | 130 | - | - | - | 1.022 | - | 5.0 | Trace | - | - | - |
|  | V4 | 130 | Trace | - | - | 1.020 | - | 5.0 | Trace | - | - | - |
|  | V5 | 130 | - | - | - | 1.012 | - | 5.0 | Trace | - | - | - |
|  | V6 | 130 | - | - | - | 1.020 | - | 6.0 | Trace | - | - | - |
|  | V7 | 130 | - | - | - | 1.012 | - | 5.0 | Trace | - | - | - |
|  | V8 | 130 | - | - | - | 1.012 | - | 5.0 | Trace | - | - | - |
|  | V9 | 130 | - | - | - | 1.012 | - | 5.0 | Trace | - | - | - |
|  | V10 | 130 | - | - | - | 1.026 | - | 5.0 | Trace | - | - | - |
| Third Round | V1 | 130 | - | - | - | 1.016 | - | 5.0 | Trace | - | - | - |
|  | V2 | 130 | - | - | - | 1.012 | - | 5.0 | Trace | - | - | - |
|  | V3 | 130 | - | - | - | 1.012 | - | 9.0 | Trace | - | - | - |
|  | V4 | 130 | Trace | - | - | 1.028 | - | 5.0 | Trace | - | - | - |
|  | V5 | 130 | - | - | - | 1.020 | - | 9.0 | Trace | - | - | - |
|  | V6 | 130 | - | - | - | 1.020 | - | 5.0 | Trace | - | - | - |
|  | V7 | 130 | - | - | - | 1.014 | - | 5.0 | Trace | - | - | - |
|  | V8 | 130 | - | - | - | 1.016 | - | 5.0 | Trace | - | - | - |
|  | V9 | 130 | - | - | - | 1.020 | - | 5.0 | Trace | - | - | - |
|  | V10 | 130 | - | - | - | 1.016 | - | 5.0 | Trace | - | - | - |
